# Exocytosis of Alphaherpesvirus Virions, Light Particles, and Glycoproteins Uses Constitutive Secretory Mechanisms

**DOI:** 10.1128/mBio.00820-16

**Published:** 2016-06-07

**Authors:** Ian B. Hogue, Julian Scherer, Lynn W. Enquist

**Affiliations:** Department of Molecular Biology and Princeton Neuroscience Institute, Princeton University, Princeton, New Jersey, USA

## Abstract

Many molecular and cell biological details of the alphaherpesvirus assembly and egress pathway remain unclear. Recently we developed a live-cell fluorescence microscopy assay of pseudorabies virus (PRV) exocytosis, based on total internal reflection fluorescence (TIRF) microscopy and a virus-encoded pH-sensitive fluorescent probe. Here, we use this assay to distinguish three classes of viral exocytosis in a nonpolarized cell type: (i) trafficking of viral glycoproteins to the plasma membrane, (ii) exocytosis of viral light particles, and (iii) exocytosis of virions. We find that viral glycoproteins traffic to the cell surface in association with constitutive secretory Rab GTPases and exhibit free diffusion into the plasma membrane after exocytosis. Similarly, both virions and light particles use these same constitutive secretory mechanisms for egress from infected cells. Furthermore, we show that viral light particles are distinct from cellular exosomes. Together, these observations shed light on viral glycoprotein trafficking steps that precede virus particle assembly and reinforce the idea that virions and light particles share a biogenesis and trafficking pathway.

## IMPORTANCE

The alphaherpesviruses, including the important human pathogens herpes simplex virus 1 (HSV-1), HSV-2, and varicella-zoster virus (VZV), are among the few viruses that have evolved to exploit the mammalian nervous system. These viruses typically cause mild recurrent herpetic or zosteriform lesions but can also cause debilitating herpes encephalitis, more frequently in very young, old, immunocompromised, or nonnatural hosts. Importantly, many of the molecular and cellular mechanisms of viral assembly and egress remain unclear. This study addresses the trafficking of viral glycoproteins to the plasma membrane, exocytosis of light particles, and exocytosis of virions. Trafficking of glycoproteins affects immune evasion and pathogenesis and may precede virus particle assembly. The release of light particles may also contribute to immune evasion and pathogenesis. Finally, exocytosis of virions is important to understand, as this final step in the virus replication cycle produces infectious extracellular particles capable of spreading to the next round of host cells.

## INTRODUCTION

Pseudorabies virus (PRV; suid herpesvirus 1) is an important veterinary pathogen related to the human alphaherpesviruses herpes simplex virus 1 (HSV-1), HSV-2, and varicella-zoster virus (VZV). The alphaherpesvirus assembly and egress pathway is understood in broad strokes, but many of the molecular and cell biological details of individual steps in the pathway remain unknown. Capsid assembly occurs in the nucleus, and capsids exit the nucleus by budding through the nuclear membranes and releasing an unenveloped capsid into the cytoplasm. Capsids, together with tegument proteins, then recruit microtubule motors for transport through the cytoplasm (1) to the site of secondary envelopment. Viral membrane proteins are produced in the secretory pathway and also traffic to the site of secondary envelopment. The intracellular trafficking routes that viral membrane proteins take to the site of secondary envelopment remain unclear. Based mainly on colocalization with *trans*-Golgi network (TGN) markers, many studies support the idea that alphaherpesvirus secondary envelopment occurs directly at TGN membranes (2). However, it is clear that viral membrane proteins do traffic to the plasma membrane, and many of them contain endocytosis motifs (e.g., YXXΦ motifs) that direct them into the endocytic pathway (3, 4).

This plasma membrane trafficking of viral membrane proteins may be important for at least two reasons. First, the diverse functions of viral membrane proteins may include interactions with extracellular or plasma membrane-localized viral or host factors. For example, the viral glycoprotein complex gE/gI has IgG Fc receptor activity and has been shown to promote endocytosis of antibodies targeting viral glycoprotein gD (5). In addition, glycoprotein gM has been shown to mediate the internalization of several viral and host membrane proteins from the cell surface, including gD and gH/gL, thereby modulating cell-cell fusion (6). Second, viral glycoproteins may traffic to the site of secondary envelopment via the plasma membrane and endocytic pathway (7).

Once viral structural components converge at the membranes of secondary envelopment, nascent virions are finally assembled by budding into the lumen of these membranes, producing an enveloped virion inside an intracellular vesicle. This virion-in-a-vesicle then traffics to the plasma membrane, and the virion exits by exocytosis.

In addition to assembly of infectious virions, the alphaherpesviruses produce a multitude of noninfectious “light particles,” or “L-particles” (8). These particles are similar in size to virions and contain viral tegument and membrane proteins but do not contain virus capsids or genomes. Although both viral light particles and cell-derived extracellular microvesicles (i.e., exosomes) deliver biologically active molecules (e.g., proteins and RNA) between cells, their relationship remains unclear (9, 10).

Several laboratories investigated the Rab GTPases involved in the alphaherpesvirus assembly and egress pathway using small interfering RNA (siRNA) knockdown and collectively found that each of the following Rabs is required for efficient HSV-1 production (7, 11–13): Rab1 is involved in endoplasmic reticulum (ER)-to-Golgi apparatus traffic; Rab6 is involved in TGN-to-plasma membrane traffic and constitutive exocytosis (14); Rab5 is involved in endocytosis and traffic through early endosomes; and Rab11 is involved in traffic through and exocytosis from recycling endosomes. However, a major caveat to these types of loss-of-function studies is that perturbing intracellular membrane trafficking produces many confounding effects on multiple steps in the assembly and egress pathway. Consequently, knockdown of any of these Rab proteins generally disrupts the assembly of infectious virions, so these approaches are not ideal to molecularly dissect particular steps in the assembly and egress pathway.

Recently, we described a novel live-cell fluorescence microscopy assay of viral exocytosis (15). This method takes advantage of live-cell total internal reflection fluorescence (TIRF) microscopy to selectively image dynamics near the plasma membrane of adherent cells and a novel pH-sensitive fluorescent probe to unambiguously identify the location and moment of viral exocytosis. We found that exocytosis of particles is associated with Rab6, Rab8, and Rab11; however, we were unable to distinguish between virions and light particles (15).

In the present study, we expand on these previous findings to distinguish three classes of viral exocytosis events: (i) trafficking of viral glycoproteins, (ii) exocytosis of light particles, and (iii) exocytosis of virions. We find that viral glycoproteins traffic to the cell surface in association with Rab6a, Rab8a, and Rab11a and exhibit free diffusion into the plasma membrane after exocytosis. Furthermore, we find that both virions and light particles use the same Rab6a, Rab8a, and Rab11a constitutive secretory mechanisms and that viral light particles are distinct from cellular exosomes, reinforcing the idea that virions and light particles share a biogenesis and trafficking pathway.

## RESULTS

### Live-cell fluorescence microscopy distinguishes three classes of viral exocytosis events.

We recently described a live-cell fluorescence microscopy assay of viral exocytosis (15). To visualize exocytosis of virus glycoproteins and particles, we constructed recombinant PRV strains expressing gM-pHluorin, a fluorescent protein biosensor that consists of superecliptic pHluorin (16) inserted into the first extracellular domain of glycoprotein M (gM; *UL10*). We previously validated the single-step replication of these PRV recombinants, as well as the expression, membrane topology, and virion incorporation of gM-pHluorin (15).

**FIG 1:**
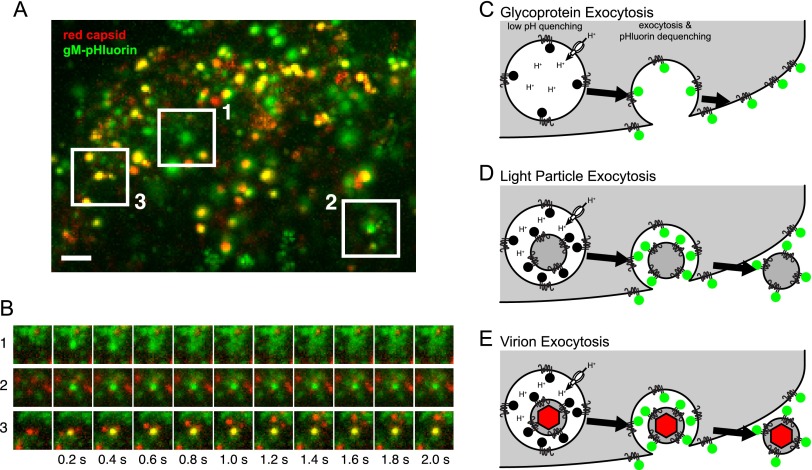
Three classes of viral exocytosis. (A) Cells infected with PRV expressing gM-pHluorin (green) and a red capsid tag were imaged beginning at 4.5 h postinfection. Image is a maximum-difference projection, to accentuate areas where gM-pHluorin intensity increases rapidly, projected over a 13-min time course. Boxed areas indicate 3 classes of viral exocytosis events. Scale bar represents 2 µm. (B) Still images corresponding to the boxed areas in panel A. (C to E) Schematics to aid in interpretation of viral exocytosis classes. (C) Schematic of glycoprotein exocytosis, corresponding to box 1 in panel A. gM-pHluorin in a secretory vesicle is quenched in its acidic lumen (black circles). Upon exocytosis, pHluorin is exposed to neutral extracellular medium, becomes fluorescent (green circles), and diffuses into the plasma membrane. (D) Schematic of light-particle exocytosis, corresponding to box 2 in panel A. Upon exocytosis, gM-pHluorin incorporated into light particles becomes fluorescent (green circles) and remains punctate. (E) Schematic of virion exocytosis, corresponding to box 3 in panel A. gM-pHluorin incorporated into a virion is quenched (black circles), but the red capsid tag is not (red hexagon). Upon exocytosis, gM-pHluorin becomes fluorescent (green circles) and remains colocalized with the red capsid.

Both secretory and endocytic organelles are acidified (pH ~5.5 to 6.5) by the action of vacuolar ATPase (vATPase) proton pumps. With a reported pK_a_ of 7.2 (16), the pHluorin moiety is strongly quenched in the lumen of these intracellular membranes. Upon exocytosis, the pHluorin moiety is exposed to the extracellular medium (pH ~7.4) and becomes brightly fluorescent (Fig. 1).

We infected PK15 cells, a nonpolarized transformed porcine epithelial cell line, with PRV 483, expressing gM-pHluorin and a monomeric red fluorescent protein (mRFP)-tagged capsid protein (red capsid), and imaged infected cells by TIRF microscopy beginning at 4.5 h postinfection. We readily distinguish three classes of exocytosis events, as follows: (i) exocytosis of vesicles containing gM-pHluorin, followed by the rapid diffusion of gM-pHluorin into the plasma membrane (Fig. 1A, box 1, and C); (ii) exocytosis of light particles containing gM-pHluorin but not capsids (Fig. 1A, box 2, and D); and (iii) exocytosis of virions containing both gM-pHluorin and capsid tags (Fig. 1A, box 3, and E). We previously noted the existence of these three types of exocytosis events (15), and here, we more fully distinguish and analyze each of these classes.

### Viral glycoprotein exocytosis is associated with Rab6, Rab8, and Rab11.

To determine which Rab GTPases are associated with the trafficking of viral glycoproteins to the plasma membrane in the absence of virus particles, we transduced PK15 cells with adenovirus vectors expressing mCherry fluorescent protein-tagged Rab proteins, infected them with PRV 486 or PRV 1026, and imaged them by TIRF microscopy beginning at 4.5 h after PRV infection. We identified exocytosis events where gM-pHluorin rapidly diffuses into the plasma membrane and measured the fluorescence intensities over a time course before and after exocytosis. We aligned the fluorescence time course data of many exocytosis events to a common time 0 based on the moment of gM-pHluorin dequenching and calculated an ensemble average of fluorescence intensities.

**FIG 2:**
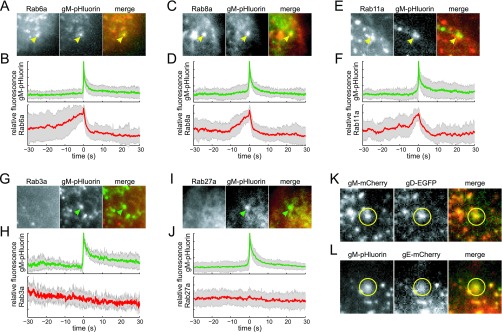
Glycoprotein exocytosis. Cells were transduced to express mCherry-tagged Rab proteins, infected with PRV expressing gM-pHluorin, and imaged beginning at 4.5 h after PRV infection. Exocytosis events corresponding to glycoprotein vesicles were selected for analysis (see Fig. 1C). (A and B) Rab6a is associated with gM-pHluorin exocytosis. (C and D) Rab8a is associated with gM-pHluorin exocytosis. (E and F) Rab11a is associated with gM-pHluorin exocytosis. (G and H) Rab3a is not associated with gM-pHluorin exocytosis. (I and J) Rab27a is not associated with gM-pHluorin exocytosis. (A, C, E, G, and I) Still images at the moment of exocytosis show colocalization (yellow arrowheads) or lack of colocalization (green arrowheads) with the indicated proteins. All images are of a 7.2-µm area. (B, D, F, H, and J) Fluorescence divided by fluorescence at time 0 (*f*/*f*_0_) ensemble averages of gM-pHluorin (top, green lines) and indicated Rab protein (bottom, red lines) over a 60-s time course. Shaded areas represent standard deviations. (K) Glycoproteins gM and gD can undergo exocytosis together (yellow circles). (L) Glycoproteins gM and gE can undergo exocytosis together (yellow circles).

We found that Rab6a, Rab8a, and Rab11a are associated with trafficking of viral glycoproteins to the plasma membrane (Fig. 2A to F). Prior to exocytosis, the ensemble average mCherry-Rab fluorescence gradually increases, representing the arrival of secretory vesicles to the site of exocytosis. Upon exocytosis, gM-pHluorin fluorescence spikes, but it decays rapidly as the gM-pHluorin diffuses out of the site of exocytosis into the surrounding plasma membrane. Likewise, the mCherry-Rab fluorescence decays rapidly, consistent with GTP hydrolysis and dissociation of the Rab proteins from the membrane (Fig. 2B, D, and F). In contrast, Rab3a and Rab27a were not associated with exocytosis in PK15 cells (Fig. 2G to J), consistent with our previous observations that these Rabs are not associated with exocytosis of particles in this cell type (15).

We additionally found that multiple viral glycoproteins can cotraffic to the plasma membrane with gM (Fig. 2K and L). We coinfected PK15 cells with PRV 347 and PRV GS1236, expressing mCherry-tagged gM (gM-mCherry) and glycoprotein D (gD; *US6*) fused to enhanced green fluorescent protein (EGFP) (gD-EGFP), respectively, and observed these glycoproteins undergoing exocytosis together (Fig. 2K). Similarly, we transduced PK15 cells with an adenovirus vector expressing PRV glycoprotein E (gE; *US8*) tagged with mCherry (gE-mCherry) and subsequently infected the cells with PRV 486, expressing gM-pHluorin. Although gE-mCherry predominantly localized to intracellular membranes (data not shown), we did occasionally observe gE-mCherry cotrafficking and undergoing exocytosis with gM-pHluorin (Fig. 2L). It is possible that since gE is overexpressed relative to its binding partner glycoprotein I (gI), only a minor fraction of gE can be transported to the plasma membrane under our experimental conditions. Altogether, these observations indicate that multiple viral glycoproteins traffic via this Rab6a, Rab8a, and Rab11a secretory pathway.

### Viral glycoproteins exhibit free diffusion in the plasma membrane after exocytosis.

**FIG 3:**
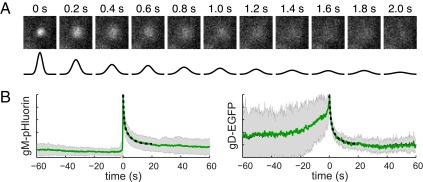
Diffusion coefficients of viral glycoproteins in the plasma membrane. (A) Still images of a representative gM-pHluorin exocytosis event over time. Diffusion coefficient of gM-pHluorin was measured by fitting a Gaussian curve (black lines) to the spatial distribution of fluorescence at each time point. All images are of a 3.6-µm area. (B) Relative fluorescence (*f*/*f*_0_) ensemble averages of gM-pHluorin or gD-EGFP over a 120-s time course. Diffusion coefficients were estimated by fitting the fluorescence decay curve to a biexponential model (dashed lines). Shaded areas represent standard deviations.

To characterize the diffusion of viral glycoproteins after exocytosis, we identified glycoprotein exocytosis events in PK15 cells infected with PRV 483, expressing gM-pHluorin. We measured the diffusion coefficient *D* of gM-pHluorin in the plasma membrane by fitting a Gaussian function to the spatial distribution of fluorescence intensity during the first 2 s after exocytosis (Fig. 3A). At the instant of vesicle fusion, gM-pHluorin fluorescence is concentrated in a diffraction-limited spot that is fit well by a narrow Gaussian curve (Fig. 3A, 0 s). For free diffusion, the square of the width of this Gaussian curve increases linearly with time, proportionally to the diffusion coefficient *D* (see Materials and Methods). We analyzed 14 gM-pHluorin exocytosis events in this manner, yielding an average diffusion coefficient of *D* = 1.3 × 10^−9^ cm^2^/s. Previous reports, using a variety of different methods, measured similar diffusion coefficients for other viral glycoproteins, including vesicular stomatitis virus G protein (*D* = 1.3 × 10^−9^ cm^2^/s [17]; *D* = ~2 × 10^−9^ cm^2^/s [18]) and influenza virus hemagglutinin (*D* = ~1.5 × 10^−9^ cm^2^/s [18]), as well as cellular transmembrane proteins (19).

As an alternative method, we also estimated diffusion coefficients by fitting the ensemble average fluorescence decay curves of many exocytosis events. We identified glycoprotein exocytosis events in PK15 cells infected with PRV 483, expressing gM-pHluorin, or PRV GS1236, expressing gD-EGFP. The fluorescence intensity during the first 10 s after exocytosis was best fit by a biexponential model with a fast half-life time constant (τ_1/2_ = 0.32 s for gM-pHluorin; τ_1/2_ = 0.31 s for gD-EGFP) and a slow component (τ_1/2_ = 2.4 s for gM-pHluorin; τ_1/2_ = 1.7 s for gD-EGFP) describing the rate of fluorescence decay (Fig. 3B). The fast component corresponds to a diffusion coefficient of *D* = 1.0 × 10^−9^ cm^2^/s for both gM-pHluorin and gD-EGFP, consistent with our measurements described above, and the slow component suggests that glycoprotein diffusion may be hindered by membrane heterogeneity and protein-protein interactions at longer timescales.

### Exocytosis of light particles is associated with Rab6, Rab8, and Rab11.

**FIG 4:**
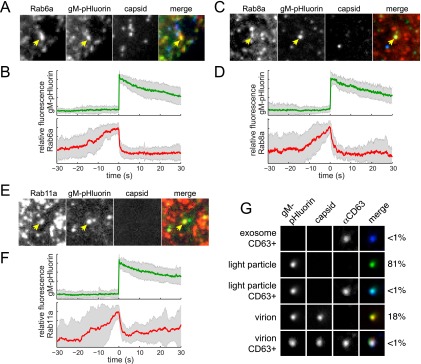
Light-particle exocytosis. Cells were transduced to express mCherry-tagged Rab proteins, infected with PRV expressing gM-pHluorin and a cyan capsid tag, and imaged at 4.5 to 5 h after PRV infection. Exocytosis events corresponding to light particles were selected for analysis (see Fig. 1D). (A and B) Rab6a is associated with light particle exocytosis. (C and D) Rab8a is associated with light-particle exocytosis. (E and F) Rab11a is associated with light-particle exocytosis. (A, C, and E) Still images at the moment of exocytosis show colocalization between gM-pHluorin and indicated Rab protein (yellow arrowheads) but not a cyan capsid tag. All images are of a 7.2-µm area. (B, D, and F) Relative fluorescence (*f*/*f*_0_) ensemble averages of gM-pHluorin (top, green lines) and indicated Rab protein (bottom, red lines) over a 60-s time course. Shaded areas represent standard deviations. (G) Particles in infected-cell supernatants were imaged to detect gM-pHluorin (green), red capsid tag, and immunofluorescence staining of CD63, a cellular exosome marker (blue). Images depict single representative particles. The percentage of particles in each category, out of a total of *n* = 1,627 particles, is indicated.

To determine whether the same Rab GTPases are associated with light-particle exocytosis, we transduced and infected PK15 cells and imaged them by TIRF microscopy, as described above. To distinguish light particles and virions, we either coinfected the cells with PRV 486 and PRV 950, which express gM-pHluorin and mTurquoise2 fluorescent protein-tagged capsid protein (cyan capsid), respectively, or infected them with PRV 1026, which expresses both fluorescent protein fusions. We performed three-color TIRF microscopy to detect mCherry-Rab proteins, gM-pHluorin, and cyan capsids, identified exocytosis events where gM-pHluorin remains punctate after exocytosis, and classified puncta based on absence of the cyan capsid tag. We also analyzed exocytosis events from a *UL25*-null mutant (PRV 495), which assembles defective capsids that fail to exit the nucleus (20, 21) and, therefore, produces only light particles and not capsid-containing virions (15). Since we observed no differences in dynamics or Rab association with either approach, we calculated ensemble averages by pooling three-color TIRF microscopy data and two-color *UL25*-null data. We found that Rab6a, Rab8a, and Rab11a are associated with exocytosis of viral light particles (Fig. 4A to F).

### Light particles are distinct from CD63-positive exosomes.

To distinguish between viral light particles and cellular microvesicles (i.e., exosomes), we performed immunofluorescence labeling of freshly prepared infected cell supernatants using an anti-CD63 antibody. The cellular protein CD63 is a late endosome/multivesicular body marker that is highly enriched in exosomes. We readily detected CD63-positive puncta in mock-infected supernatants (data not shown) and PRV 483-infected supernatants (Fig. 4G), consistent with cellular exosomes. In PRV 483-infected supernatants, 81% of puncta contained gM-pHluorin but no capsid, consistent with light particles. Eighteen percent of puncta contained both gM-pHluorin and capsids, consistent with virions (Fig. 4G). Interestingly, puncta with capsids (virions) incorporated approximately 3 times more gM-pHluorin than puncta without (light particles). Importantly, only 0.2% of gM-pHluorin-positive puncta contained detectable amounts of CD63, suggesting that light particles and virions are distinct from CD63-positive exosomes (Fig. 4G).

### Virion exocytosis is associated with Rab6, Rab8, and Rab11.

**FIG 5:**
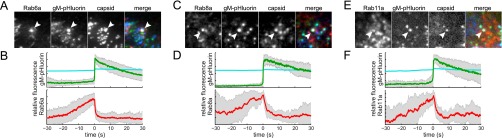
Virion exocytosis. Cells were transduced to express mCherry-tagged Rab proteins, infected with PRV expressing gM-pHluorin and a cyan capsid tag, and imaged at 4.5 to 5 h after PRV infection. Exocytosis events corresponding to virions were selected for analysis (see Fig. 1E). (A and B) Rab6a is associated with virion exocytosis. (C and D) Rab8a is associated with virion exocytosis. (E and F) Rab11a is associated with virion exocytosis. (A, C, and E) Still images at the moment of exocytosis show colocalization between gM-pHluorin, cyan capsid tag, and indicated Rab protein (white arrowheads). All images are of a 7.2-µm area. (B, D, and F) Relative fluorescence (*f*/*f*_0_) ensemble averages of gM-pHluorin (top, green line), cyan capsid (top, cyan line), and indicated Rab protein (bottom, red line) over a 60-s time course. Shaded areas represent standard deviations.

Finally, to determine whether these same Rab GTPases are associated with virion exocytosis, we again transduced and infected PK15 cells and imaged them by TIRF microscopy. As with our analysis of light particles, described above, we performed three-color TIRF microscopy to detect mCherry-Rab proteins, gM-pHluorin, and cyan capsids, identifying exocytosis of virions that contain the cyan capsid tag. Previously, we performed limited three-color TIRF microscopy and observed Rab6a associated with individual exocytosis events of virions containing fluorescent capsids (15). Here, we better quantify this Rab6a association by calculating ensemble averages over many exocytosis events, and additionally, we find that Rab8a and Rab11a are associated with exocytosis of virions containing fluorescent capsids (Fig. 5).

## DISCUSSION

The assembly and egress of alphaherpesvirus particles is a highly dynamic process driven by viral and host cell molecular machinery. In this study, we expanded on previous work to distinguish three classes of viral exocytosis events: exocytosis of viral glycoproteins, light particles, and virions (Fig. 1). We found that Rab6a, Rab8a, and Rab11a were dynamically associated with all three types of viral exocytosis, implicating these same constitutive secretory mechanisms in multiple steps in the virus replication cycle. Other secretory Rab proteins that govern Ca^2+^-regulated exocytosis, Rab3a and Rab27a, do not appear to be involved in this nonpolarized cell type (Fig. 2) (15), but it remains to be seen whether alphaherpesviruses use this regulated secretory pathway in polarized, professional secretory cell types, such as neurons.

### Relationship to betaherpesvirus assembly and egress.

In comparison to the alphaherpesviruses, human cytomegalovirus (HCMV), a betaherpesvirus, causes drastic reorganization of intracellular membranes into a structure referred to as the “perinuclear body,” “virus factory,” or “assembly compartment” (reviewed in references 2 and [Bibr B22][Bibr B23][Bibr B24]). The assembly compartment appears to be organized into concentric layers around the microtubule organizing center (MTOC), with endosomal markers in the center, surrounded in turn by TGN, Golgi and ER membranes, all straddled by the enlarged kidney-shaped nucleus (25). Both the TGN and endosomal markers appear to colocalize at the assembly compartment (26), including Rab6 (27) and Rab11 (28, 29). HCMV tegument and membrane proteins also colocalize at the assembly compartment, and capsids associated with wrapping membranes are visible by electron microscopy; therefore, it is thought that this structure is the site of HCMV secondary envelopment. HCMV and human herpesvirus 6 (HHV-6), another betaherpesvirus, are reported to bud into membranes containing both TGN and endosomal markers and incorporate both TGN and endosomal markers into infectious virions (26, 30). In particular, the late endosome/multivesicular body and exosome marker, CD63, is associated with assembly and egress of both HCMV and HHV-6 (26, 30), implicating the exosome biogenesis and release pathway.

Similar to the betaherpesviruses, HSV-1 capsids associated with wrapping membranes containing an exogenous endocytic cargo were observed in a recent study, indicating that the membranes of alphaherpesvirus secondary envelopment may be at least partially derived by endocytosis from the plasma membrane (7). We show that Rab6, Rab8, and Rab11 are each associated with PRV secretory vesicles. Together, Rab6 and Rab8 regulate the constitutive exocytosis of post-Golgi secretory vesicles (31), and Rab8 and Rab11 together regulate the constitutive exocytosis of recycling endosomes (32). Thus, alphaherpesviruses may also assemble at some confluence of secretory and endosomal membranes, albeit with much less extensive spatial reorganization of these intracellular membranes than occurs with HCMV. However, in contrast to several observations in the betaherpesvirus literature (26, 30, 33, 34), we find that PRV exocytosis is not associated with Rab3 or Rab27 (Fig. 2) (15), and the particles do not incorporate detectable amounts of CD63 (Fig. 4). Thus, there may yet be fundamental differences between the alpha- and betaherpesvirus assembly and egress pathways.

## MATERIALS AND METHODS

### Cells and viruses.

PK15 cells and 293A cells were cultured in Dulbecco’s modified Eagle’s medium (DMEM) supplemented with 10% fetal bovine serum (FBS) and penicillin-streptomycin, as previously described (15). For live-cell TIRF microscopy, PK15 cells were seeded onto serum-coated 25-mm glass coverslips (Fisher) or 35-mm glass-bottom cell culture dishes (Mat-Tek). After incubating for several hours to allow cells to adhere, PK15 cells were transduced with adenovirus vectors, incubated overnight, and then infected with PRV recombinants.

The adenovirus vectors expressing mCherry-tagged Rab3a, Rab6a, Rab8a, Rab11a, and Rab27a were previously described (15). An adenovirus vector expressing gE-mCherry was constructed by PCR amplification of the gE-coding sequence from PRV strain Becker, followed by Gateway recombination upstream from the mCherry coding sequence in pAd/CMV/V5-DEST (Invitrogen). The fusion junction between gE and mCherry is as follows (the C-terminal sequence of gE is shown in plain font, an 8-residue linker is in boldface, and the N-terminal sequence of mCherry is italicized): …ASRLLNARPA**TTLYTKVV***VSKGEEDNMA*…. The cloned gE sequence was confirmed by DNA sequencing, and the functionality of the gE-mCherry fusion was validated by rescuing anterograde axonal transport of a gE-null PRV mutant in superior cervical ganglion neurons (J. Scherer and L. W. Enquist, unpublished data). All adenovirus vectors were propagated on complementing 293A cells, cell-associated virus was harvested in serum-free DMEM, and the transduction efficiency of the resulting stocks was estimated by visualizing fluorescent protein expression in noncomplementing PK15 cells.

All PRV recombinants (Table 1) are derivatives of PRV Becker. PRV 486 expresses gM-pHluorin, PRV 483 expresses gM-pHluorin and mRFP-VP26, and PRV 495 expresses gM-pHluorin and mRFP-VP26 and contains a *UL25*-null mutation (15). PRV 950 expresses mTurquoise2-VP26 (15, 35). PRV 1026, expressing gM-pHluorin and mTurquoise2-VP26, was constructed by coinfecting PRV 483 and PRV 950 and screening plaques for loss of mRFP-VP26 and gain of mTurquoise2-VP26 by fluorescence microscopy. PRV GS1236 expresses gD-EGFP and was kindly provided by Greg A. Smith at Northwestern University (36).

**TABLE 1:** Pseudorabies virus recombinants

Virus	Description
PRV 486	gM-pHluorin
PRV 483	gM-pHluorin, mRFP-VP26 (red capsid)
PRV 495	gM-pHluorin, mRFP-VP26 (red capsid), *UL25* null
PRV 950	mTurquoise2-VP26 (cyan capsid)
PRV 1026	gM-pHluorin, mTurquoise2-VP26 (cyan capsid)
PRV GS1236	gD-EGFP

### Virus particle immunofluorescence.

Confluent PK15 cell monolayers were infected with PRV 483 at a multiplicity of infection (MOI) of 5 PFU per cell or mock infected, incubated at 37°C for 1 h, thoroughly rinsed with Hanks’ balanced salt solution (HBSS), and incubated overnight in DMEM containing 2% FBS. Cell supernatants were harvested at ~14 h postinfection, pipetted onto 35-mm glass-bottom Mat-Tek dishes, and incubated briefly to allow particles to nonspecifically adhere to the glass without drying. The dishes were then incubated for 15 min in a 1:100 dilution of monoclonal anti-CD63 antibody (BD Pharmingen), followed by 15 min in a 1:200 dilution of anti-mouse Alexa Fluor 647 secondary antibody (Invitrogen). Individual particles were imaged using a previously described epifluorescence microscope (37) equipped with a Plan Apo 100×/1.40 numerical aperture (NA) oil immersion objective (Nikon) and a Photometrics CoolSNAP ES2 charge-coupled device (CCD) camera. Particle fluorescence intensities were measured from 20 random fields of view using the Analyze Particles function in Fiji/ImageJ, version 1.48 (38).

### TIRF microscopy.

The two-color live-cell TIRF movies were acquired using a previously described custom-built microscope in the Princeton University Lewis-Sigler Imaging Core Facility (15). Three-color live-cell TIRF movies were acquired on a previously described Nikon N-STORM microscope in the Princeton University Molecular Biology Confocal Microscopy Facility (15), or a previously described custom-built ring-TIRF microscope in the Enquist laboratory (39). Images were prepared for publication using the following functions and plugins in Fiji/ImageJ: adjust brightness and contrast, Kalman filter (to reduce noise in time course microscopy images), and Z project (to make maximum-intensity projections). We calculated a maximum-difference projection, depicted in Fig. 1A, which emphasizes areas where the gM-pHluorin fluorescence intensity rapidly increases, as previously described (15).

### Diffusion analysis.

The diffusion of gM-pHluorin was measured by Gaussian fitting as follows. A square, 30- by 30-pixel region of interest surrounding each glycoprotein exocytosis event was selected for analysis. At each time point, the center of mass was calculated as the weighted average of the brightest 5% of pixels. The radial intensity distribution was determined by projecting image data onto the polar coordinate system around the calculated center of mass and then fitting the data to a Gaussian function, as follows: $I\left(r\right)=ae^{-r^{2}/w^{2}}+b$, where *I*(r) is the fluorescence intensity at radius *r*, *a* is the peak intensity at the center of mass, *w*^2^ represents the width of the Gaussian curve, and *b* is the background fluorescence intensity. For free diffusion, the Gaussian width *w*^2^ increases linearly over time *t*, and the diffusion coefficient *D* can be calculated from Δ*w*^2^ = 4*D*Δ*t*, as previously described (17, 19).

The diffusion of gM-pHluorin and gD-EGFP was measured by biexponential fitting as follows: A 3-pixel-radius region of interest around each glycoprotein exocytosis event was selected for analysis. The fluorescence intensity was recorded over a 10-s time course after exocytosis, and the ensemble average over many exocytosis events was calculated as described above. The resulting ensemble average fluorescence decay curve was fit to a biexponential function, $I\left(t\right)=a2^{-t/\tau_{1}}+b2^{-t/\tau_{2}}+c$, in order to determine the half-life time constants τ_1_ and τ_2_. The diffusion coefficient *D* can be estimated from the time constant according to the equation *D* = *r*^2^/4τ, where *r* is the radius of the measured region of interest (here, *r* = 360 nm) and τ is the half-life time constant (40, 41).

## References

[1] Sodeik B 2000 Mechanisms of viral transport in the cytoplasm. Trends Microbiol 8:465–472. doi:10.1016/S0966-842X(00)01824-2. 11044681

[2] Henaff D, Radtke K, Lippé R 2012 Herpesviruses exploit several host compartments for envelopment. Traffic 13:1443–1449. doi:10.1111/j.1600-0854.2012.01399.x. 22805610

[3] Favoreel HW 2006 The why’s of Y-based motifs in alphaherpesvirus envelope proteins. Virus Res 117:202–208. doi:10.1016/j.virusres.2005.11.007. 16417939

[4] Brideau AD, Enquist LW, Tirabassi RS 2000 The role of virion membrane protein endocytosis in the herpesvirus life cycle. J Clin Virol 17:69–82. doi:10.1016/S1386-6532(00)00084-6. 10942087

[5] Ndjamen B, Farley AH, Lee T, Fraser SE, Bjorkman PJ 2014 The herpes virus Fc receptor gE-gI mediates antibody bipolar bridging to clear viral antigens from the cell surface. PLoS Pathog 10:e00820-16. doi:10.1371/journal.ppat.1003961. PMC3946383 24604090

[6] Crump CM, Bruun B, Bell S, Pomeranz LE, Minson T, Browne HM 2004 Alphaherpesvirus glycoprotein M causes the relocalization of plasma membrane proteins. J Gen Virol 85:3517–3527. doi:10.1099/vir.0.80361-0. 15557225

[7] Hollinshead M, Johns HL, Sayers CL, Gonzalez-Lopez C, Smith GL, Elliott G 2012 Endocytic tubules regulated by Rab GTPases 5 and 11 are used for envelopment of herpes simplex virus. EMBO J 31:4204–4220. doi:10.1038/emboj.2012.262. 22990238 PMC3492727

[8] Szilágyi JF, Cunningham C 1991 Identification and characterization of a novel non-infectious herpes simplex virus-related particle. J Gen Virol 72:661–668. doi:10.1099/0022-1317-72-3-661. 1848601

[9] Meckes DG, Raab-Traub N 2011 Microvesicles and viral infection. J Virol 85:12844–12854. doi:10.1128/JVI.05853-11. 21976651 PMC3233125

[10] Kalamvoki M, Deschamps T 2016 Extracellular vesicles during herpes simplex virus type 1 infection: an inquire. Virol J 13:63. doi:10.1186/s12985-016-0518-2. 27048572 PMC4822280

[11] Johns HL, Gonzalez-Lopez C, Sayers CL, Hollinshead M, Elliott G 2014 Rab6 dependent post-Golgi trafficking of HSV1 envelope proteins to sites of virus envelopment. Traffic 15:157–178. doi:10.1111/tra.12134. 24152084 PMC4345966

[12] Zenner HL, Yoshimura S, Barr FA, Crump CM 2011 Analysis of Rab GTPase-activating proteins indicates that Rab1a/b and Rab43 are important for herpes simplex virus 1 secondary envelopment. J Virol 85:8012–8021. doi:10.1128/JVI.00500-11. 21680502 PMC3147948

[13] Stegen C, Yakova Y, Henaff D, Nadjar J, Duron J, Lippé R 2013 Analysis of virion-incorporated host proteins required for herpes simplex virus type 1 infection through a RNA interference screen. PLoS One 8:e00820-16. doi:10.1371/journal.pone.0053276. PMC3536771 23301054

[14] Grigoriev I, Splinter D, Keijzer N, Wulf PS, Demmers J, Ohtsuka T, Modesti M, Maly IV, Grosveld F, Hoogenraad CC, Akhmanova A 2007 Rab6 regulates transport and targeting of exocytotic carriers. Dev Cell 13:305–314. doi:10.1016/j.devcel.2007.06.010. 17681140

[15] Hogue IB, Bosse JB, Hu J-R, Thiberge SY, Enquist LW 2014 Cellular mechanisms of alpha herpesvirus egress: live cell fluorescence microscopy of pseudorabies virus exocytosis. PLoS Pathog 10:e00820-16. doi:10.1371/journal.ppat.1004535. PMC4256261 25474634

[16] Sankaranarayanan S, De Angelis D, Rothman JE, Ryan TA 2000 The use of pHluorins for optical measurements of presynaptic activity. Biophys J 79:2199–2208. doi:10.1016/S0006-3495(00)76468-X. 11023924 PMC1301110

[17] Schmoranzer J, Goulian M, Axelrod D, Simon SM 2000 Imaging constitutive exocytosis with total internal reflection fluorescence microscopy. J Cell Biol 149:23–32. doi:10.1083/jcb.149.1.23. 10747084 PMC2175105

[18] Kenworthy AK, Nichols BJ, Remmert CL, Hendrix GM, Kumar M, Zimmerberg J, Lippincott-Schwartz J 2004 Dynamics of putative raft-associated proteins at the cell surface. J Cell Biol 165:735–746. doi:10.1083/jcb.200312170. 15173190 PMC2172371

[19] Allersma MW, Wang L, Axelrod D, Holz RW 2004 Visualization of regulated exocytosis with a granule-membrane probe using total internal reflection microscopy. Mol Biol Cell 15:4658–4668. doi:10.1091/mbc.E04-02-0149. 15282339 PMC519157

[20] McNab AR, Desai P, Person S, Roof LL, Thomsen DR, Newcomb WW, Brown JC, Homa FL 1998 The product of the herpes simplex virus type 1 UL25 gene is required for encapsidation but not for cleavage of replicated viral DNA. J Virol 72:1060–1070. 9445000 10.1128/jvi.72.2.1060-1070.1998 PMC124578

[21] Klupp BG, Granzow H, Keil GM, Mettenleiter TC 2006 The capsid-associated UL25 protein of the alphaherpesvirus pseudorabies virus is nonessential for cleavage and encapsidation of genomic DNA but is required for nuclear egress of capsids. J Virol 80:6235–6246. doi:10.1128/JVI.02662-05. 16775311 PMC1488961

[B22] Alwine JC 2012 The human cytomegalovirus assembly compartment: a masterpiece of viral manipulation of cellular processes that facilitates assembly and egress. PLoS Pathog 8:e00820-16. doi:10.1371/journal.ppat.1002878. PMC3447744 23028305

[B23] Johnson DC, Baines JD 2011 Herpesviruses remodel host membranes for virus egress. Nat Rev Microbiol 9:382–394. doi:10.1038/nrmicro2559. 21494278

[B24] Tandon R, Mocarski ES 2012 Viral and host control of cytomegalovirus maturation. Trends Microbiol 20:392–401. doi:10.1016/j.tim.2012.04.008. 22633075 PMC3408842

[25] Das S, Vasanji A, Pellett PE 2007 Three-dimensional structure of the human cytomegalovirus cytoplasmic virion assembly complex includes a reoriented secretory apparatus. J Virol 81:11861–11869. doi:10.1128/JVI.01077-07. 17715239 PMC2168812

[26] Cepeda V, Esteban M, Fraile-Ramos A 2010 Human cytomegalovirus final envelopment on membranes containing both *trans*-Golgi network and endosomal markers. Cell Microbiol 12:386–404. doi:10.1111/j.1462-5822.2009.01405.x. 19888988

[27] Indran SV, Britt WJ 2011 A role for the small GTPase Rab6 in assembly of human cytomegalovirus. J Virol 85:5213–5219. doi:10.1128/JVI.02605-10. 21411515 PMC3126173

[28] Das S, Pellett PE 2011 Spatial relationships between markers for secretory and endosomal machinery in human cytomegalovirus-infected cells versus those in uninfected cells. J Virol 85:5864–5879. doi:10.1128/JVI.00155-11. 21471245 PMC3126327

[29] Krzyzaniak MA, Mach M, Britt WJ 2009 HCMV-encoded glycoprotein M (UL100) interacts with Rab11 effector protein FIP4. Traffic 10:1439–1457. doi:10.1111/j.1600-0854.2009.00967.x. 19761540 PMC4118585

[30] Mori Y, Koike M, Moriishi E, Kawabata A, Tang H, Oyaizu H, Uchiyama Y, Yamanishi K 2008 Human herpesvirus-6 induces MVB formation, and virus egress occurs by an exosomal release pathway. Traffic 9:1728–1742. doi:10.1111/j.1600-0854.2008.00796.x. 18637904 PMC2613231

[31] Grigoriev I, Yu KL, Martinez-Sanchez E, Serra-Marques A, Smal I, Meijering E, Demmers J, Peränen J, Pasterkamp RJ, van der Sluijs P, Hoogenraad CC, Akhmanova A 2011 Rab6, Rab8, and MICAL3 cooperate in controlling docking and fusion of exocytotic carriers. Curr Biol 21:967–974. doi:10.1016/j.cub.2011.04.030. 21596566

[32] Hsu VW, Prekeris R 2010 Transport at the recycling endosome. Curr Opin Cell Biol 22:528–534. doi:10.1016/j.ceb.2010.05.008. 20541925 PMC2910225

[33] Fraile-Ramos A, Cepeda V, Elstak E, van der Sluijs P 2010 Rab27a is required for human cytomegalovirus assembly. PLoS One 5:e00820-16. doi:10.1371/journal.pone.0015318. PMC2999566 21170347

[34] Homman-Loudiyi M, Hultenby K, Britt W, Söderberg-Nauclér C 2003 Envelopment of human cytomegalovirus occurs by budding into Golgi-derived vacuole compartments positive for gB, Rab 3, *trans*-Golgi network 46, and mannosidase II. J Virol 77:3191–3203. doi:10.1128/JVI.77.5.3191-3203.2003. 12584343 PMC149787

[35] Hogue IB, Bosse JB, Engel EA, Scherer J, Hu J-R, Del Rio T, Enquist LW 2015 Fluorescent protein approaches in alpha herpesvirus research. Viruses 7:5933–5961. doi:10.3390/v7112915. 26610544 PMC4664988

[36] Antinone SE, Smith GA 2006 Two modes of herpesvirus trafficking in neurons: membrane acquisition directs motion. J Virol 80:11235–11240. doi:10.1128/JVI.01441-06. 16971439 PMC1642139

[37] Taylor MP, Kratchmarov R, Enquist LW 2013 Live cell imaging of alphaherpes virus anterograde transport and spread. J Vis Exp 2013:e50723. doi:10.3791/50723. PMC3852683 23978901

[38] Schindelin J, Arganda-Carreras I, Frise E, Kaynig V, Longair M, Pietzsch T, Preibisch S, Rueden C, Saalfeld S, Schmid B, Tinevez J-Y, White DJ, Hartenstein V, Eliceiri K, Tomancak P, Cardona A 2012 Fiji: an open-source platform for biological-image analysis. Nat Methods 9:676–682. doi:10.1038/nmeth.2019. 22743772 PMC3855844

[39] Bosse JB, Hogue IB, Feric M, Thiberge SY, Sodeik B, Brangwynne CP, Enquist LW 2015 Remodeling nuclear architecture allows efficient transport of herpesvirus capsids by diffusion. Proc Natl Acad Sci U S A 112:E5725–E5733. doi:10.1073/pnas.1513876112. 26438852 PMC4620878

[40] Soumpasis DM 1983 Theoretical analysis of fluorescence photobleaching recovery experiments. Biophys J 41:95–97. doi:10.1016/S0006-3495(83)84410-5. 6824758 PMC1329018

[41] Kang M, Day CA, Kenworthy AK, DiBenedetto E 2012 Simplified equation to extract diffusion coefficients from confocal FRAP data. Traffic 13:1589–1600. doi:10.1111/tra.12008. 22984916 PMC3731631

